# Regulation of Pacing Strategy during Athletic Competition

**DOI:** 10.1371/journal.pone.0015863

**Published:** 2011-01-20

**Authors:** Jos J. de Koning, Carl Foster, Arjan Bakkum, Sil Kloppenburg, Christian Thiel, Trent Joseph, Jacob Cohen, John P. Porcari

**Affiliations:** 1 Faculty of Human Movement Sciences, Research Institute MOVE, VU University Amsterdam, Amsterdam, The Netherlands; 2 Department of Exercise and Sport Science, University of Wisconsin La Crosse, La Crosse, Wisconsin, United States of America; 3 Department of Sportmedicine, Goethe-Universität, Frankfurt, Germany; Universidad Europea de Madrid, Spain

## Abstract

**Background:**

Athletic competition has been a source of interest to the scientific community for many years, as a surrogate of the limits of human ambulatory ability. One of the remarkable things about athletic competition is the observation that some athletes suddenly reduce their pace in the mid-portion of the race and drop back from their competitors. Alternatively, other athletes will perform great accelerations in mid-race (surges) or during the closing stages of the race (the endspurt). This observation fits well with recent evidence that muscular power output is regulated in an anticipatory way, designed to prevent unreasonably large homeostatic disturbances.

**Principal Findings:**

Here we demonstrate that a simple index, the product of the momentary Rating of Perceived Exertion (RPE) and the fraction of race distance remaining, the Hazard Score, defines the likelihood that athletes will change their velocity during simulated competitions; and may effectively represent the language used to allow anticipatory regulation of muscle power output.

**Conclusions:**

These data support the concept that the muscular power output during high intensity exercise performance is actively regulated in an anticipatory manner that accounts for both the momentary sensations the athlete is experiencing as well as the relative amount of a competition to be completed.

## Introduction

The observation of changes in the pattern of velocity during competition has led to interest in pacing strategy during athletic competitions [1]–[6]. There have been at least three basic types of pacing strategies identified (positive, negative and even pacing strategy), which depend on the duration of the event and the consequences of slowing with a loss in power output [2], [7]–[9]. There is some agreement that pacing strategy is organized in an anticipatory way designed not only to optimize performance but also to prevent unreasonably large homeostatic disturbances during the exercise bout [5], [6]. There is also some agreement that different elements of the physiologic response to exercise are involved in regulating pacing strategy. Just as the driver of a race car might monitor different gauges during a race, and pay more attention to one gauge during a short race and another during a longer race; it appears that intramuscular substrate/metabolite changes [1], [10]–[11] are more likely to be determining of changes in muscular power output during shorter (1–30 min) competitions, with body core (or brain) temperature being more central during mid-duration (20–120 min) events [12]–[17], and the availability of carbohydrate as an oxidizable substrate being critical in longer (>90 min) events [18]–[19]. While the effect of these different physiological regulators doubtless overlaps, their net input appears to be integrated by the conscious brain using the Rating of Perceived Exertion (RPE) [20]–[23]. In a recently proposed model, Tucker (6) suggested that changes in the homeostatic status, reflected by the momentary RPE, allows alteration of pacing strategy (power output) in both an anticipatory and responsive manner based on pre-exercise expectations and peripheral feedback from different physiological sensors.

It has been shown that RPE increases in an approximately linear manner as a function of the proportion of an event completed [24]–[28], and has scalar characteristics when plotted against percentage of the exercise task completed (time or distance). Together these data suggest that the athlete is continuously comparing how they feel at any moment in a competition with how they expected to feel at that moment. If their RPE is larger than expected for that point in the event, then power output (e.g. running speed) will decrease, even if it means giving up on the competition. If RPE is less than expected, then power output will increase. The process of controlling muscular power output via RPE apparently occurs continuously throughout an exercise bout and almost certainly takes into account the amount of distance remaining to be covered, as well as the momentary value of RPE [6], [23]. If an athlete approaches critical levels of homeostatic disturbance (which can vary depending on the length of a race; e.g. pH disturbances in a race of 2 min duration, temperature increases in a race of 90 min duration, or limited availability of carbohydrate as an oxidizable substrate in a race of 180 min duration) before nearing the end-point of a competition, a progressive reduction in power output may take place [8]. This protective mechanism is apparently quite effective as it is very unusual to see an athlete continue until muscle contractions entirely fail or to run to hyperthermic collapse. Indeed, perhaps the only time the regulatory mechanism completely fails is in pathological conditions such as those typified by neuromuscular diseases (e.g. myasthenia gravis, McArdle disease), musculoskeletal disease (acute muscle or tendon ruptures), cardiovascular disease (e.g. ventricular arrhythmia, myocardial infarction triggered by exertion, exertion related congestive heart failure, peripheral vascular disease) or respiratory disease (exertional bronchospasm). Likewise, as the distance remaining becomes sufficiently small, the athlete may choose to use their remaining energetic reserves in an endspurt, regardless of the level of homeostatic disturbance. Effectively the exerciser must compute the hazard (e.g. danger of a competitively decisive competitive collapse or health related collapse) if a certain pace is maintained during the early or mid-portion part of an event versus the ability to achieve their competitive goal.

**Figure 1:**
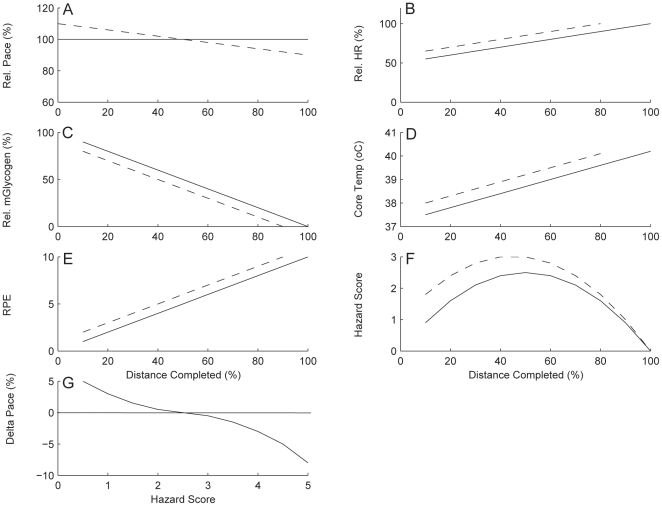
Relation between the Hazard Score and the change in pace. Schematic pace (A), heart rate (HRmax%) (B), muscle glycogen store (C), core temperature (D), Rating of Perceived Exertion (RPE) (E) and Hazard Score (F) as a function of percent distance completed of a hypothetical athlete performing a race with a fast start strategy and with an even strategy and the resulting relation between the Hazard Score and the change in pace during the race (G).

On a conceptual level it can be hypothesized that an athlete performing an event in a fast start manner compared to an even paced race will have higher RPE values throughout the entire race (Figure 1 A, E). The higher RPE will be the result of the sub-conscious detection of, for instance, a higher heart rate [29], a faster depletion of glycogen stores [30] and/or an earlier rise in core temperature [17] (Figure 1B-D). The higher RPE in the fast start event will result in a higher ‘hazard of catastrophic collapse’, compared to the ‘even’ or ‘normal’ paced trial. This ‘hazard’ could be seen as something that could be dangerous for the physiological system. However, the ‘hazard’ must also be understood from the perspective of the amount of the event remaining. Accordingly we conceptualized a measure for the ‘hazard’ as the product of the momentary RPE with the fraction of the remaining distance; the ‘Hazard Score’ (Figure 1F). Our hypothesis is that the value of the Hazard Score is associated with the ability to accelerate during the race or to the need to reduce the speed to values at which the homeostatic disturbances stay within acceptable limits (Figure 1G). We sought to determine whether simple integration of the momentary RPE by the percentage of distance remaining (e.g. Hazard Score) could adequately explain within event variations in velocity by competitive athletes.

## Methods

To accomplish this, we integrated the velocity and RPE data of 9 separate experiments, in which either cyclists or runners completed competitive simulations in the laboratory, in events that required from 4 to 60 minutes. The individual studies were approved by the Institutional Review Board for the Protection of Human Subjects of the University of Wisconsin-La Crosse, and each subject provided written informed consent prior to participation. Some of the original data have previously been published [24] but are analyzed with a different purpose here, and some represent previously unpublished data. All of the individual exercise studies involved closed loop time trials, with the subjects instructed to finish the complete test in the smallest possible time. Some of the studies were performed on a racing cycle with computerized measurement of power output, velocity and distance. Others were performed on a motor driven treadmill. During these competitive simulations, all of the subjects were well-trained sub-elite athletes who were task habituated to the test event. In some of the events, the subjects were free to self pace their effort, with the intent of minimizing the time to complete the event. In others we imposed, for experimental reasons, either a starting strategy that was more aggressive than normally chosen by the athlete or which included a mid-race increase in pace, or environmental challenges. In all studies, the Rating of Perceived Exertion was measured using the Category Ratio version of the RPE scale [31]. We integrated the mean value for each series of experiments (10–12 subjects in each series) with reference to predicting changes in velocity during the event in terms of the ‘Hazard Score’, calculated as the product of the momentary RPE and the fraction of the event remaining at the same point. As a general principle, measures of RPE were obtained at approximately 10% of full distance increments during each competitive simulation.

## Results

**Figure 2:**
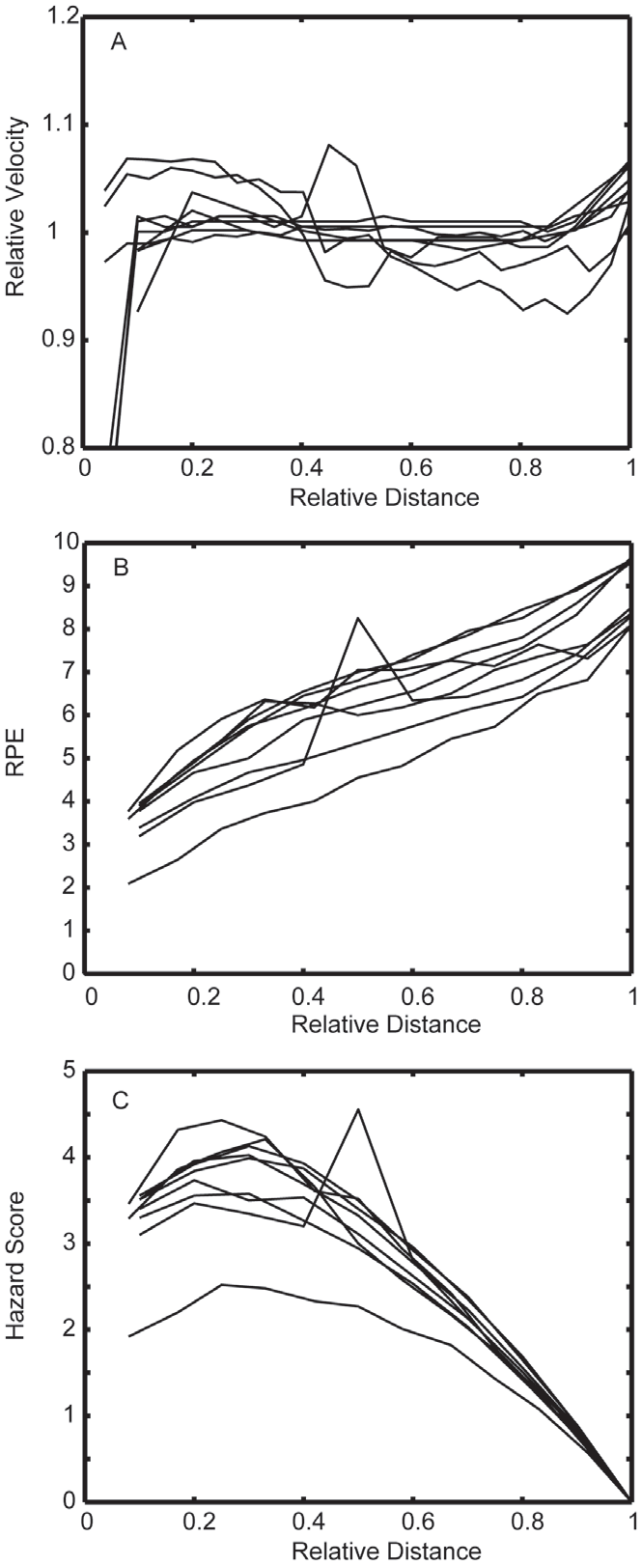
Velolcity, RPE and the Hazard Score. Changes in velocity (A), Rate of Perceived Exertion (RPE) (B) and the Hazard Score (C) in 9 competitive simulations in running or cycling events that required from 4 to 60 minutes.

Serial changes in velocity, RPE and the computed Hazard Score in all nine experiments are presented in Figure 2. The growth of RPE in relation to the proportional distance matched previous observations [6], [24], [26], [28]. The value for the Hazard Score reached peak values usually during the first half of each event, with the exception of experiments during which mid-race surges were performed. Since the computation of the Hazard Score necessarily includes a zero value for percent of distance remaining at the end of the race, the score necessarily decreases to zero at the conclusion of the event.

**Figure 3:**
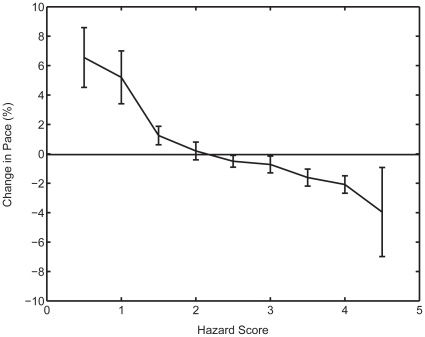
The acceleration/deceleration of athletes as function of the Hazard score.

When changes in velocity within each event were analyzed in terms of the Hazard Score, there was, as hypothesized, a regular relationship, with low values of the Hazard Score being associated with increases in running or cycling velocity (a positive value for change in pace), and high values being associated with decelerations (a negative value for change in pace) (Figure 3). There was a significant likelihood that the change in velocity would be positive (acceleration) when the Hazard Score was less than 1.5, negative when the Hazard Score was greater than 3 and unchanged when the Hazard Score was between 1–3.

## Discussion

In this analysis, we have demonstrated that the tendency of athletes to change pace during competitive simulations is related both to how they feel momentarily (RPE) and to how much of the event remains. The calculation of a simple index combining these two predictors (the Hazard Score), which represents the hazard of a competitively catastrophic collapse faced by the athlete, allows a remarkably accurate prediction of subsequent behavior. Although athletes rarely completely collapse during competition, there are enough examples (e.g. the collapse of Englishman Jim Peters during the Commonwealth Games marathon in 1954) to support the concept that there is a ‘limit’ to which humans can push themselves which can cause catastrophic collapse even in a highly trained athlete. Although this type of analysis should be tested during actual competition, the clarity of results presented here suggests a very simple explanation of how the decision making process regarding distribution of effort during competition (e.g. very heavy exercise) is made.

**Figure 4:**
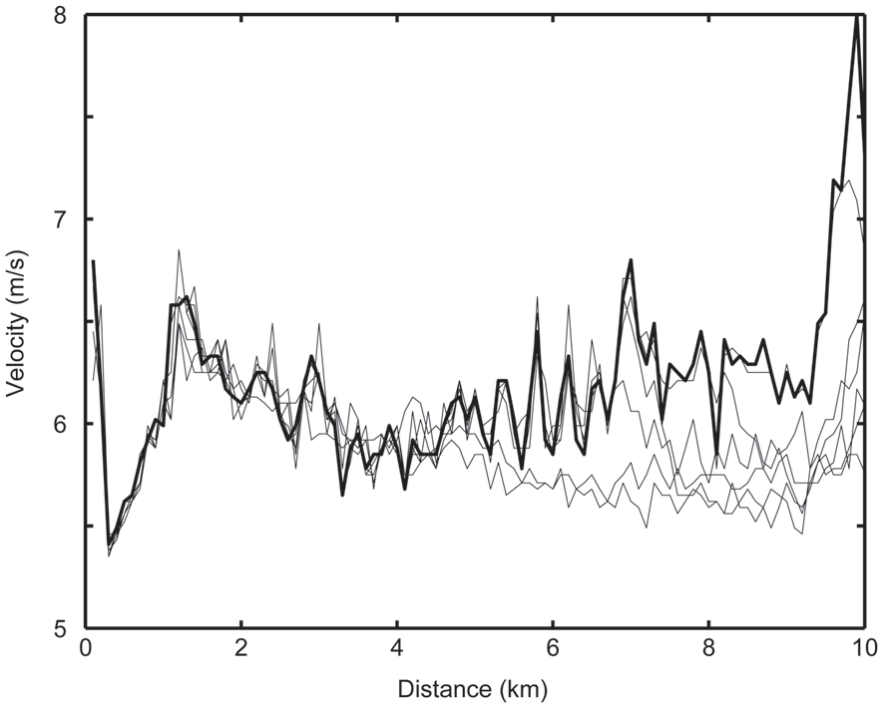
Velocity of the winner (bold) and the runners successively 5 places further back (e.g., 5^th^, 10^th^, 15^th^) from the Beijing Olympic men's 10 km race.

**Figure 5:**
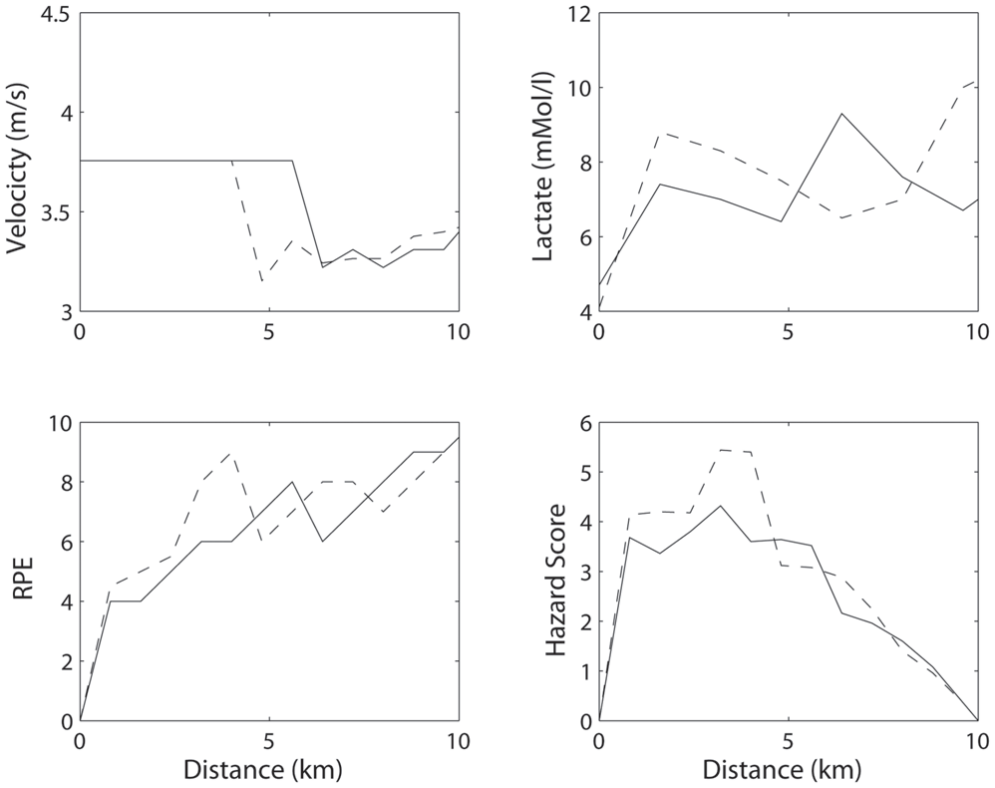
Example of a subject running two 10 km races on a treadmill. The pace during the first 4 km was set and could be varied after this point. The predictor of the slowdown at 4 km during a run at a ‘bad day’ (broken line) is the high Hazard Score in the 3–4 km interval, which reflects larger homeostatic disturbances.

Until experimental tests of this hypothesis can be made, it may be instructive to evaluate data from high level competitions to provide a real-world test of this concept. In Figure 4 we present publicly available (www.iaaf.org) data from the Beijing Olympic men's 10 km race, with the running velocity of the winner plotted (bold) and runners successively 5 places further back noted. First, it is evident that the overall pace slowed prior to 2 km (when we would have predicted high hazard scores to emerge). Second, it is evident that many of the runners dropped off the pace (e.g. ran slower than the eventual winner) at approximately the mid-point of the event and just at the moment the eventual winner began a series of accelerations (e.g. increasing RPE and thus the hazard score). Third, it is evident that during the endspurt, a period of low hazard score because of the small distance remaining, the winner simply ran faster than the remaining competitors. From this, it appears that runners who drop off in the middle portions of the race must have been confronted with an unacceptably large hazard score (e.g homeostatic disturbance) and, faced with the choice of slowing their pace or not finishing, chose to reduce their pace. Similarly, the actions of the eventual winner beginning at approximately mid race, at a time when the hazard score might be expected to be declining, was to increase velocity, which would have increased the hazard score of his competitors to the point where they dropped out of contention for winning the race. A limitation of the Olympic 10 km data is that we have no RPE or physiological variables of the athletes during their race. However, a typical example of a subject running multiple 10 km races on a treadmill in the laboratory illustrates the line of our reasoning (Figure 5). To mimic a real race the subject was forced to run at a set velocity for the first 4 km, as if he was running ’in the pack’. After 4 km he was free to vary his pace. Velocity, RPE and blood lactate concentration were measured and consequently the Hazard Score was computed. During his run on a ‘bad day’ (broken line), compared with a better performance (solid line), the RPE and lactate were running high early in the race as was the Hazard Score. The predictor of the slowdown at 4 km is the high Hazard Score in the 3–4 km interval, which reflects larger homeostatic disturbances.

The results of this study, in which the likelihood of changing velocity during a competitive simulation are related to a simple index of the momentary RPE and the percentage of the event remaining suggest the fundamental strategy by which athletes regulate their effort during competition. This suggests the possibility of correlative studies where the magnitude of homeostatic disturbances that contribute to the RPE can be understood as they change during the course of the race.
